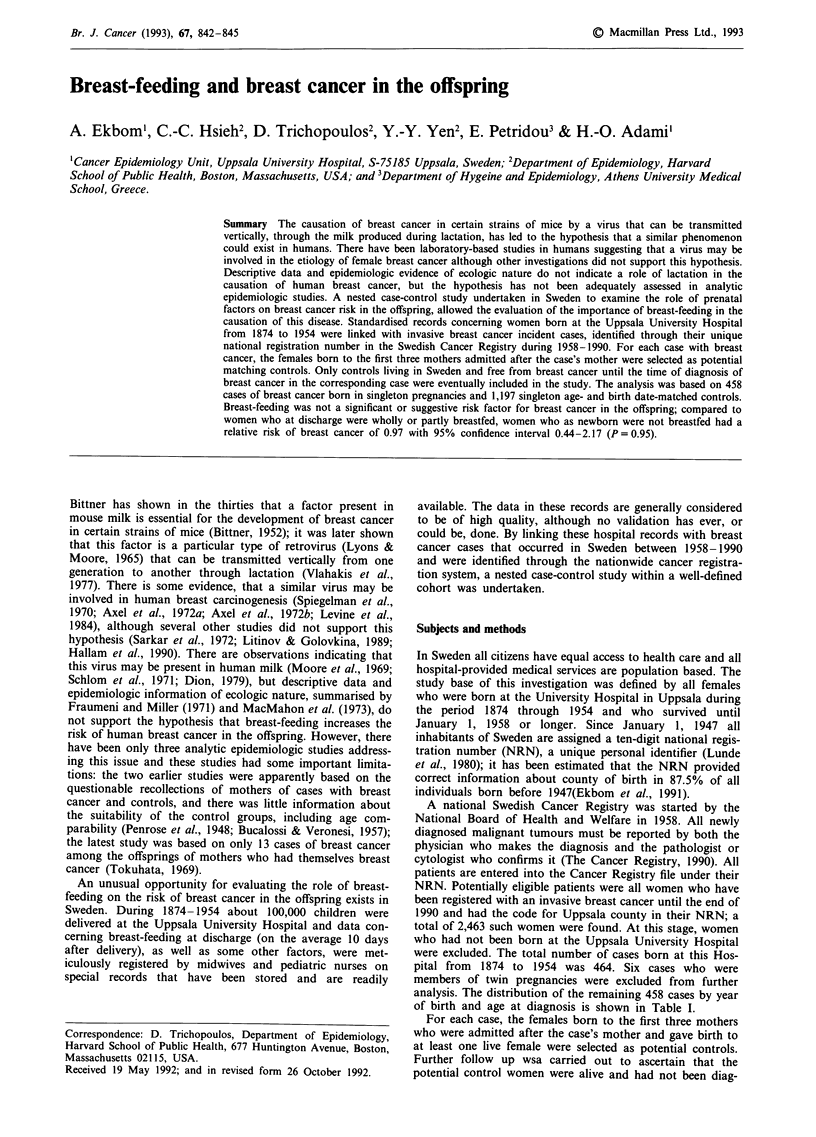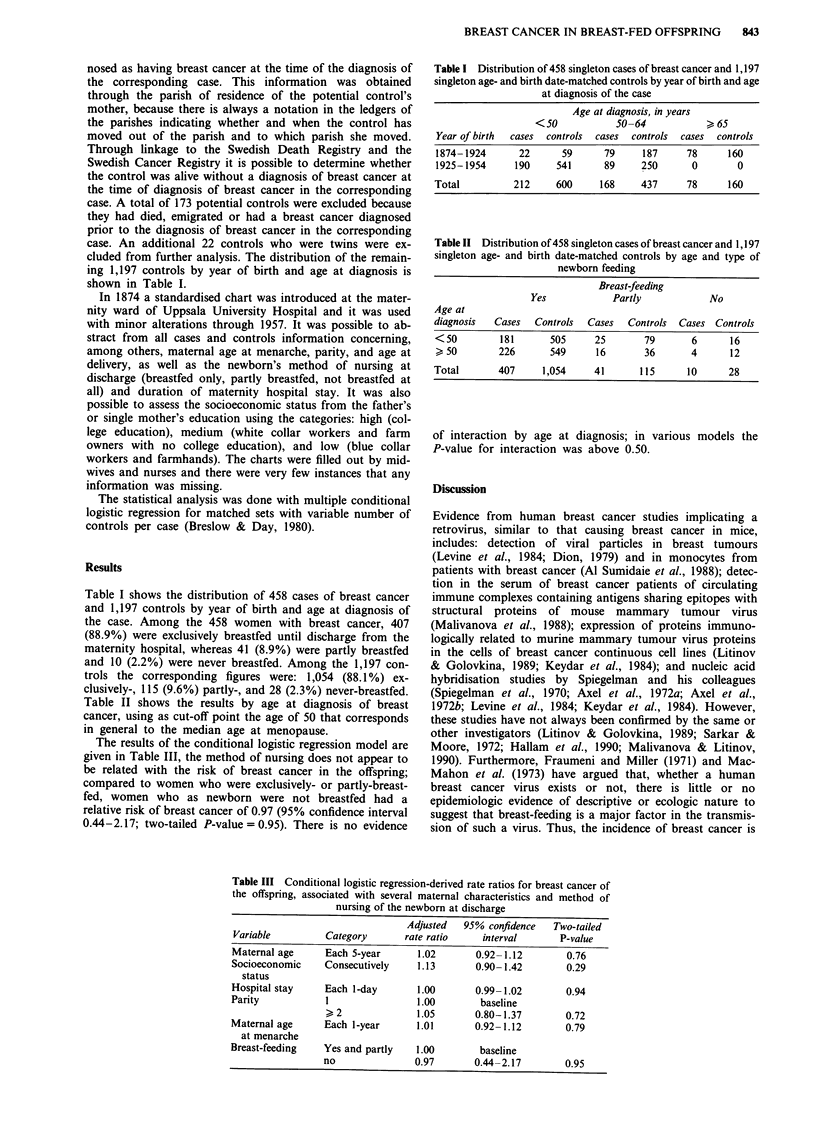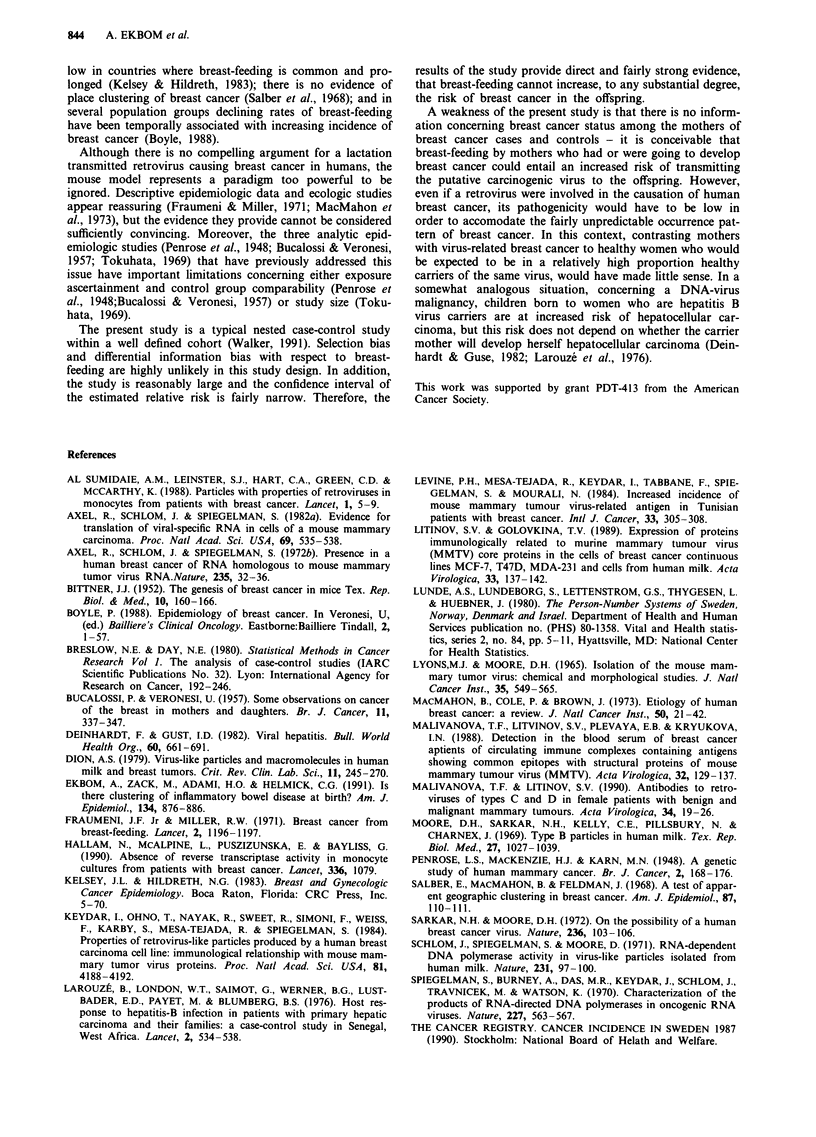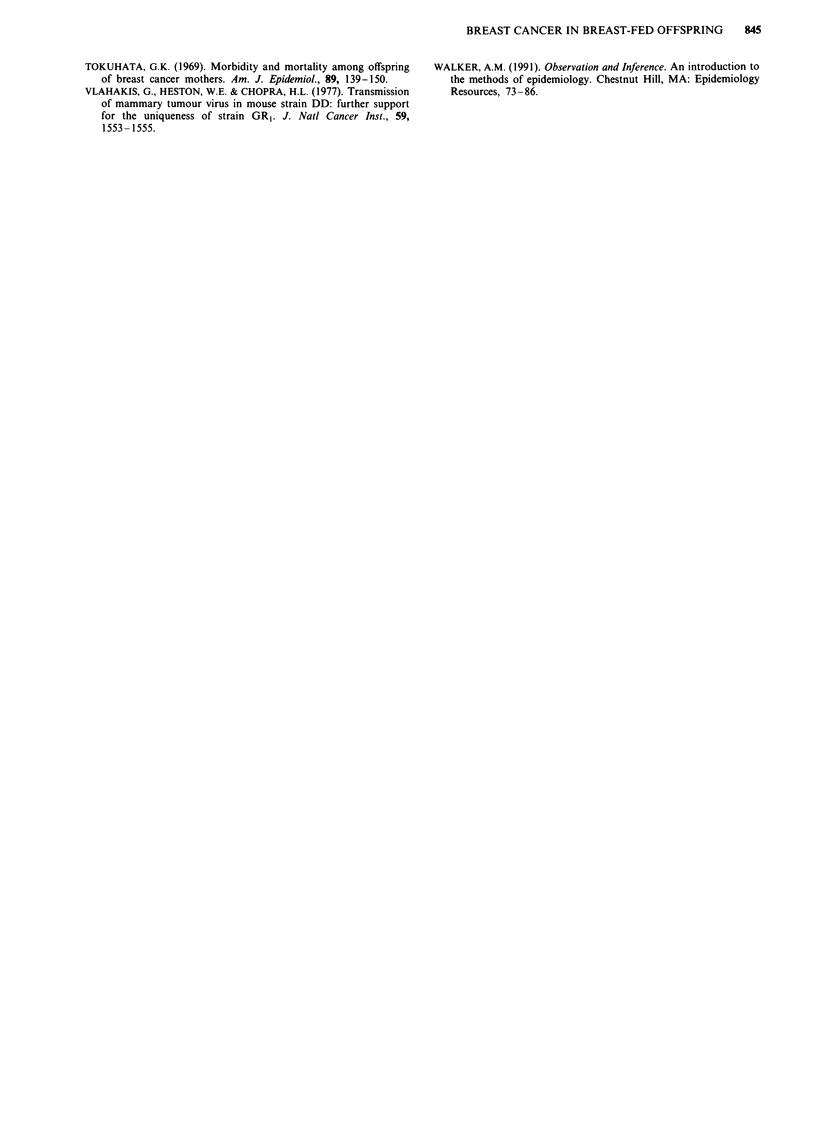# Breast-feeding and breast cancer in the offspring.

**DOI:** 10.1038/bjc.1993.154

**Published:** 1993-04

**Authors:** A. Ekbom, C. C. Hsieh, D. Trichopoulos, Y. Y. Yen, E. Petridou, H. O. Adami

**Affiliations:** Cancer Epidemiology Unit, Uppsala University Hospital, Sweden.

## Abstract

The causation of breast cancer in certain strains of mice by a virus that can be transmitted vertically, through the milk produced during lactation, has led to the hypothesis that a similar phenomenon could exist in humans. There have been laboratory-based studies in humans suggesting that a virus may be involved in the etiology of female breast cancer although other investigations did not support this hypothesis. Descriptive data and epidemiologic evidence of ecologic nature do not indicate a role of lactation in the causation of human breast cancer, but the hypothesis has not been adequately assessed in analytic epidemiologic studies. A nested case-control study undertaken in Sweden to examine the role of prenatal factors on breast cancer risk in the offspring, allowed the evaluation of the importance of breast-feeding in the causation of this disease. Standardised records concerning women born at the Uppsala University Hospital from 1874 to 1954 were linked with invasive breast cancer incident cases, identified through their unique national registration number in the Swedish Cancer Registry during 1958-1990. For each case with breast cancer, the females born to the first three mothers admitted after the case's mother were selected as potential matching controls. Only controls living in Sweden and free from breast cancer until the time of diagnosis of breast cancer in the corresponding case were eventually included in the study. The analysis was based on 458 cases of breast cancer born in singleton pregnancies and 1,197 singleton age- and birth date-matched controls. Breast-feeding was not a significant or suggestive risk factor for breast cancer in the offspring; compared to women who at discharge were wholly or partly breastfed, women who as newborn were not breastfed had a relative risk of breast cancer of 0.97 with 95% confidence interval 0.44-2.17 (P = 0.95).


Br. J. Cancer (1993), 67, 842-845 © Macmillan Press Ltd., 1993

### Breast-feeding and breast cancer in the offspring

A. Ekbom[1], C.-C. Hsieh[2], D. Trichopoulos[2], Y.-Y. Yen[2], E. Petridou[3] & H.-O. Adami[1]

[1]*Cancer Epidemiology Unit, Uppsala University Hospital, S-75185 Uppsala, Sweden;* [2]*Department of Epidemiology, Harvard School of Public Health, Boston, Massachusetts, USA; and* [3]*Department of Hygeine and Epidemiology, Athens University Medical School, Greece.*

**Summary** The causation of breast cancer in certain strains of mice by a virus that can be transmitted vertically, through the milk produced during lactation, has led to the hypothesis that a similar phenomenon could exist in humans. There have been laboratory-based studies in humans suggesting that a virus may be involved in the etiology of female breast cancer although other investigations did not support this hypothesis. Descriptive data and epidemiologic evidence of ecologic nature do not indicate a role of lactation in the causation of human breast cancer, but the hypothesis has not been adequately assessed in analytic epidemiologic studies. A nested case-control study undertaken in Sweden to examine the role of prenatal factors on breast cancer risk in the offspring, allowed the evaluation of the importance of breast-feeding in the causation of this disease. Standardised records concerning women born at the Uppsala University Hospital from 1874 to 1954 were linked with invasive breast cancer incident cases, identified through their unique national registration number in the Swedish Cancer Registry during 1958–1990. For each case with breast cancer, the females born to the first three mothers admitted after the case's mother were selected as potential matching controls. Only controls living in Sweden and free from breast cancer until the time of diagnosis of breast cancer in the corresponding case were eventually included in the study. The analysis was based on 458 cases of breast cancer born in singleton pregnancies and 1,197 singleton age- and birth date-matched controls. Breast-feeding was not a significant or suggestive risk factor for breast cancer in the offspring; compared to women who at discharge were wholly or partly breastfed, women who as newborn were not breastfed had a relative risk of breast cancer of 0.97 with 95% confidence interval 0.44–2.17 ($P = 0.95$).

Bittner has shown in the thirties that a factor present in mouse milk is essential for the development of breast cancer in certain strains of mice (Bittner, 1952); it was later shown that this factor is a particular type of retrovirus (Lyons & Moore, 1965) that can be transmitted vertically from one generation to another through lactation (Vlahakis *et al.*, 1977). There is some evidence, that a similar virus may be involved in human breast carcinogenesis (Spiegelman *et al.*, 1970; Axel *et al.*, 1972*a*; Axel *et al.*, 1972*b*; Levine *et al.*, 1984), although several other studies did not support this hypothesis (Sarkar *et al.*, 1972; Litinov & Golovkina, 1989; Hallam *et al.*, 1990). There are observations indicating that this virus may be present in human milk (Moore *et al.*, 1969; Schlom *et al.*, 1971; Dion, 1979), but descriptive data and epidemiologic information of ecologic nature, summarised by Fraumeni and Miller (1971) and MacMahon *et al.* (1973), do not support the hypothesis that breast-feeding increases the risk of human breast cancer in the offspring. However, there have been only three analytic epidemiologic studies addressing this issue and these studies had some important limitations: the two earlier studies were apparently based on the questionable recollections of mothers of cases with breast cancer and controls, and there was little information about the suitability of the control groups, including age comparability (Penrose *et al.*, 1948; Bucalossi & Veronesi, 1957); the latest study was based on only 13 cases of breast cancer among the offsprings of mothers who had themselves breast cancer (Tokuhata, 1969).

An unusual opportunity for evaluating the role of breast-feeding on the risk of breast cancer in the offspring exists in Sweden. During 1874–1954 about 100,000 children were delivered at the Uppsala University Hospital and data concerning breast-feeding at discharge (on the average 10 days after delivery), as well as some other factors, were meticulously registered by midwives and pediatric nurses on special records that have been stored and are readily available. The data in these records are generally considered to be of high quality, although no validation has ever, or could be, done. By linking these hospital records with breast cancer cases that occurred in Sweden between 1958–1990 and were identified through the nationwide cancer registration system, a nested case-control study within a well-defined cohort was undertaken.

Correspondence: D. Trichopoulos, Department of Epidemiology, Harvard School of Public Health, 677 Huntington Avenue, Boston, Massachusetts 02115, USA.
Received 19 May 1992; and in revised form 26 October 1992.

### Subjects and methods

In Sweden all citizens have equal access to health care and all hospital-provided medical services are population based. The study base of this investigation was defined by all females who were born at the University Hospital in Uppsala during the period 1874 through 1954 and who survived until January 1, 1958 or longer. Since January 1, 1947 all inhabitants of Sweden are assigned a ten-digit national registration number (NRN), a unique personal identifier (Lunde *et al.*, 1980); it has been estimated that the NRN provided correct information about county of birth in 87.5% of all individuals born before 1947(Ekbom *et al.*, 1991).

A national Swedish Cancer Registry was started by the National Board of Health and Welfare in 1958. All newly diagnosed malignant tumours must be reported by both the physician who makes the diagnosis and the pathologist or cytologist who confirms it (The Cancer Registry, 1990). All patients are entered into the Cancer Registry file under their NRN. Potentially eligible patients were all women who have been registered with an invasive breast cancer until the end of 1990 and had the code for Uppsala county in their NRN; a total of 2,463 such women were found. At this stage, women who had not been born at the Uppsala University Hospital were excluded. The total number of cases born at this Hospital from 1874 to 1954 was 464. Six cases who were members of twin pregnancies were excluded from further analysis. The distribution of the remaining 458 cases by year of birth and age at diagnosis is shown in Table I.

For each case, the females born to the first three mothers who were admitted after the case's mother and gave birth to at least one live female were selected as potential controls. Further follow up wsa carried out to ascertain that the potential control women were alive and had not been diagnosed as having breast cancer at the time of the diagnosis of the corresponding case. This information was obtained through the parish of residence of the potential control's mother, because there is always a notation in the ledgers of the parishes indicating whether and when the control has moved out of the parish and to which parish she moved. Through linkage to the Swedish Death Registry and the Swedish Cancer Registry it is possible to determine whether the control was alive without a diagnosis of breast cancer at the time of diagnosis of breast cancer in the corresponding case. A total of 173 potential controls were excluded because they had died, emigrated or had a breast cancer diagnosed prior to the diagnosis of breast cancer in the corresponding case. An additional 22 controls who were twins were excluded from further analysis. The distribution of the remaining 1,197 controls by year of birth and age at diagnosis is shown in Table I.

In 1874 a standardised chart was introduced at the maternity ward of Uppsala University Hospital and it was used with minor alterations through 1957. It was possible to abstract from all cases and controls information concerning, among others, maternal age at menarche, parity, and age at delivery, as well as the newborn's method of nursing at discharge (breastfed only, partly breastfed, not breastfed at all) and duration of maternity hospital stay. It was also possible to assess the socioeconomic status from the father's or single mother's education using the categories: high (college education), medium (white collar workers and farm owners with no college education), and low (blue collar workers and farmhands). The charts were filled out by midwives and nurses and there were very few instances that any information was missing.

The statistical analysis was done with multiple conditional logistic regression for matched sets with variable number of controls per case (Breslow & Day, 1980).

### Results

Table I shows the distribution of 458 cases of breast cancer and 1,197 controls by year of birth and age at diagnosis of the case. Among the 458 women with breast cancer, 407 (88.9%) were exclusively breastfed until discharge from the maternity hospital, whereas 41 (8.9%) were partly breastfed and 10 (2.2%) were never breastfed. Among the 1,197 controls the corresponding figures were: 1,054 (88.1%) exclusively-, 115 (9.6%) partly-, and 28 (2.3%) never-breastfed. Table II shows the results by age at diagnosis of breast cancer, using as cut-off point the age of 50 that corresponds in general to the median age at menopause.

The results of the conditional logistic regression model are given in Table III, the method of nursing does not appear to be related with the risk of breast cancer in the offspring; compared to women who were exclusively- or partly-breastfed, women who as newborn were not breastfed had a relative risk of breast cancer of 0.97 (95% confidence interval 0.44–2.17; two-tailed $P$-value = 0.95). There is no evidence

**Table I** Distribution of 458 singleton cases of breast cancer and 1,197 singleton age- and birth date-matched controls by year of birth and age at diagnosis of the case

| | *Age at diagnosis, in years* | | | | | |
|---|---|---|---|---|---|---|
| | *<50* | | *50–64* | | *≥65* | |
| *Year of birth* | *cases* | *controls* | *cases* | *controls* | *cases* | *controls* |
| 1874–1924 | 22 | 59 | 79 | 187 | 78 | 160 |
| 1925–1954 | 190 | 541 | 89 | 250 | 0 | 0 |
| Total | 212 | 600 | 168 | 437 | 78 | 160 |

**Table II** Distribution of 458 singleton cases of breast cancer and 1,197 singleton age- and birth date-matched controls by age and type of newborn feeding

| | *Breast-feeding* | | | | | |
|---|---|---|---|---|---|---|
| | *Yes* | | *Partly* | | *No* | |
| *Age at diagnosis* | *Cases* | *Controls* | *Cases* | *Controls* | *Cases* | *Controls* |
| <50 | 181 | 505 | 25 | 79 | 6 | 16 |
| ≥50 | 226 | 549 | 16 | 36 | 4 | 12 |
| Total | 407 | 1,054 | 41 | 115 | 10 | 28 |

of interaction by age at diagnosis; in various models the $P$-value for interaction was above 0.50.

### Discussion

Evidence from human breast cancer studies implicating a retrovirus, similar to that causing breast cancer in mice, includes: detection of viral particles in breast tumours (Levine *et al.*, 1984; Dion, 1979) and in monocytes from patients with breast cancer (Al Sumidaie *et al.*, 1988); detection in the serum of breast cancer patients of circulating immune complexes containing antigens sharing epitopes with structural proteins of mouse mammary tumour virus (Malivanova *et al.*, 1988); expression of proteins immunologically related to murine mammary tumour virus proteins in the cells of breast cancer continuous cell lines (Litinov & Golovkina, 1989; Keydar *et al.*, 1984); and nucleic acid hybridisation studies by Spiegelman and his colleagues (Spiegelman *et al.*, 1970; Axel *et al.*, 1972*a*; Axel *et al.*, 1972*b*; Levine *et al.*, 1984; Keydar *et al.*, 1984). However, these studies have not always been confirmed by the same or other investigators (Litinov & Golovkina, 1989; Sarkar & Moore, 1972; Hallam *et al.*, 1990; Malivanova & Litinov, 1990). Furthermore, Fraumeni and Miller (1971) and MacMahon *et al.* (1973) have argued that, whether a human breast cancer virus exists or not, there is little or no epidemiologic evidence of descriptive or ecologic nature to suggest that breast-feeding is a major factor in the transmission of such a virus. Thus, the incidence of breast cancer is

**Table III** Conditional logistic regression-derived rate ratios for breast cancer of the offspring, associated with several maternal characteristics and method of nursing of the newborn at discharge

| *Variable* | *Category* | *Adjusted rate ratio* | *95% confidence interval* | *Two-tailed P-value* |
|---|---|---|---|---|
| Maternal age | Each 5-year | 1.02 | 0.92–1.12 | 0.76 |
| Socioeconomic status | Consecutively | 1.13 | 0.90–1.42 | 0.29 |
| Hospital stay | Each 1-day | 1.00 | 0.99–1.02 | 0.94 |
| Parity | 1 | 1.00 | baseline | |
| | ≥2 | 1.05 | 0.80–1.37 | 0.72 |
| Maternal age at menarche | Each 1-year | 1.01 | 0.92–1.12 | 0.79 |
| Breast-feeding | Yes and partly | 1.00 | baseline | |
| | no | 0.97 | 0.44–2.17 | 0.95 |

low in countries where breast-feeding is common and prolonged (Kelsey & Hildreth, 1983); there is no evidence of place clustering of breast cancer (Salber *et al.*, 1968); and in several population groups declining rates of breast-feeding have been temporally associated with increasing incidence of breast cancer (Boyle, 1988).

Although there is no compelling argument for a lactation transmitted retrovirus causing breast cancer in humans, the mouse model represents a paradigm too powerful to be ignored. Descriptive epidemiologic data and ecologic studies appear reassuring (Fraumeni & Miller, 1971; MacMahon *et al.*, 1973), but the evidence they provide cannot be considered sufficiently convincing. Moreover, the three analytic epidemiologic studies (Penrose *et al.*, 1948; Bucalossi & Veronesi, 1957; Tokuhata, 1969) that have previously addressed this issue have important limitations concerning either exposure ascertainment and control group comparability (Penrose *et al.*, 1948;Bucalossi & Veronesi, 1957) or study size (Tokuhata, 1969).

The present study is a typical nested case-control study within a well defined cohort (Walker, 1991). Selection bias and differential information bias with respect to breast-feeding are highly unlikely in this study design. In addition, the study is reasonably large and the confidence interval of the estimated relative risk is fairly narrow. Therefore, the results of the study provide direct and fairly strong evidence, that breast-feeding cannot increase, to any substantial degree, the risk of breast cancer in the offspring.

A weakness of the present study is that there is no information concerning breast cancer status among the mothers of breast cancer cases and controls – it is conceivable that breast-feeding by mothers who had or were going to develop breast cancer could entail an increased risk of transmitting the putative carcinogenic virus to the offspring. However, even if a retrovirus were involved in the causation of human breast cancer, its pathogenicity would have to be low in order to accomodate the fairly unpredictable occurrence pattern of breast cancer. In this context, contrasting mothers with virus-related breast cancer to healthy women who would be expected to be in a relatively high proportion healthy carriers of the same virus, would have made little sense. In a somewhat analogous situation, concerning a DNA-virus malignancy, children born to women who are hepatitis B virus carriers are at increased risk of hepatocellular carcinoma, but this risk does not depend on whether the carrier mother will develop herself hepatocellular carcinoma (Deinhardt & Guse, 1982; Larouzé *et al.*, 1976).

This work was supported by grant PDT-413 from the American Cancer Society.

### References

AL SUMIDAIE, A.M., LEINSTER, S.J., HART, C.A., GREEN, C.D. & McCARTHY, K. (1988). Particles with properties of retroviruses in monocytes from patients with breast cancer. *Lancet*, **1**, 5–9.

AXEL, R., SCHLOM, J. & SPIEGELMAN, S. (1982*a*). Evidence for translation of viral-specific RNA in cells of a mouse mammary carcinoma. *Proc. Natl Acad. Sci. USA*, **69**, 535–538.

AXEL, R., SCHLOM, J. & SPIEGELMAN, S. (1972*b*). Presence in a human breast cancer of RNA homologous to mouse mammary tumor virus RNA.*Nature*, **235**, 32–36.

BITTNER, J.J. (1952). The genesis of breast cancer in mice Tex. *Rep. Biol. & Med.*, **10**, 160–166.

BOYLE, P. (1988). Epidemiology of breast cancer. In Veronesi, U, (ed.) *Bailliere's Clinical Oncology*. Eastborne:Bailliere Tindall, **2**, 1–57.

BRESLOW, N.E. & DAY, N.E. (1980). *Statistical Methods in Cancer Research Vol 1*. The analysis of case-control studies (IARC Scientific Publications No. 32). Lyon: International Agency for Research on Cancer, 192–246.

BUCALOSSI, P. & VERONESI, U. (1957). Some observations on cancer of the breast in mothers and daughters. *Br. J. Cancer*, **11**, 337–347.

DEINHARDT, F. & GUST, I.D. (1982). Viral hepatitis. *Bull. World Health Org.*, **60**, 661–691.

DION, A.S. (1979). Virus-like particles and macromolecules in human milk and breast tumors. *Crit. Rev. Clin. Lab. Sci.*, **11**, 245–270.

EKBOM, A., ZACK, M., ADAMI, H.O. & HELMICK, C.G. (1991). Is there clustering of inflammatory bowel disease at birth? *Am. J. Epidemiol.*, **134**, 876–886.

FRAUMENI, J.F. Jr & MILLER, R.W. (1971). Breast cancer from breast-feeding. *Lancet*, **2**, 1196–1197.

HALLAM, N., MCALPINE, L., PUSZIZUNSKA, E. & BAYLISS, G. (1990). Absence of reverse transcriptase activity in monocyte cultures from patients with breast cancer. *Lancet*, **336**, 1079.

KELSEY, J.L. & HILDRETH, N.G. (1983). *Breast and Gynecologic Cancer Epidemiology*. Boca Raton, Florida: CRC Press, Inc. 5–70.

KEYDAR, I., OHNO, T., NAYAK, R., SWEET, R., SIMONI, F., WEISS, F., KARBY, S., MESA-TEJADA, R. & SPIEGELMAN, S. (1984). Properties of retrovirus-like particles produced by a human breast carcinoma cell line: immunological relationship with mouse mammary tumor virus proteins. *Proc. Natl Acad. Sci. USA*, **81**, 4188–4192.

LAROUZÉ, B., LONDON, W.T., SAIMOT, G., WERNER, B.G., LUSTBADER, E.D., PAYET, M. & BLUMBERG, B.S. (1976). Host response to hepatitis-B infection in patients with primary hepatic carcinoma and their families: a case-control study in Senegal, West Africa. *Lancet*, **2**, 534–538.

LEVINE, P.H., MESA-TEJADA, R., KEYDAR, I., TABBANE, F., SPIEGELMAN, S. & MOURALI, N. (1984). Increased incidence of mouse mammary tumour virus-related antigen in Tunisian patients with breast cancer. *Intl J. Cancer*, **33**, 305–308.

LITINOV, S.V. & GOLOVKINA, T.V. (1989). Expression of proteins immunologically related to murine mammary tumour virus (MMTV) core proteins in the cells of breast cancer continuous lines MCF-7, T47D, MDA-231 and cells from human milk. *Acta Virologica*, **33**, 137–142.

LUNDE, A.S., LUNDEBORG, S., LETTENSTROM, G.S., THYGESEN, L. & HUEBNER, J. (1980). *The Person-Number Systems of Sweden, Norway, Denmark and Israel*. Department of Health and Human Services publication no. (PHS) 80-1358. Vital and Health statistics, series 2, no. 84, pp. 5–11, Hyattsville, MD: National Center for Health Statistics.

LYONS,M.J. & MOORE, D.H. (1965). Isolation of the mouse mammary tumor virus: chemical and morphological studies. *J. Natl Cancer Inst.*, **35**, 549–565.

MACMAHON, B., COLE, P. & BROWN, J. (1973). Etiology of human breast cancer: a review. *J. Natl Cancer Inst.*, **50**, 21–42.

MALIVANOVA, T.F., LITVINOV, S.V., PLEVAYA, E.B. & KRYUKOVA, I.N. (1988). Detection in the blood serum of breast cancer aptients of circulating immune complexes containing antigens showing common epitopes with structural proteins of mouse mammary tumour virus (MMTV). *Acta Virologica*, **32**, 129–137.

MALIVANOVA, T.F. & LITINOV, S.V. (1990). Antibodies to retroviruses of types C and D in female patients with benign and malignant mammary tumours. *Acta Virologica*, **34**, 19–26.

MOORE, D.H., SARKAR, N.H., KELLY, C.E., PILLSBURY, N. & CHARNEX, J. (1969). Type B particles in human milk. *Tex. Rep. Biol. Med.*, **27**, 1027–1039.

PENROSE, L.S., MACKENZIE, H.J. & KARN, M.N. (1948). A genetic study of human mammary cancer. *Br. J. Cancer*, **2**, 168–176.

SALBER, E., MACMAHON, B. & FELDMAN, J. (1968). A test of apparent geographic clustering in breast cancer. *Am. J. Epidemiol.*, **87**, 110–111.

SARKAR, N.H. & MOORE, D.H. (1972). On the possibility of a human breast cancer virus. *Nature*, **236**, 103–106.

SCHLOM, J., SPIEGELMAN, S. & MOORE, D. (1971). RNA-dependent DNA polymerase activity in virus-like particles isolated from human milk. *Nature*, **231**, 97–100.

SPIEGELMAN, S., BURNEY, A., DAS, M.R., KEYDAR, J., SCHLOM, J., TRAVNICEK, M. & WATSON, K. (1970). Characterization of the products of RNA-directed DNA polymerases in oncogenic RNA viruses. *Nature*, **227**, 563–567.

THE CANCER REGISTRY. CANCER INCIDENCE IN SWEDEN 1987 (1990). Stockholm: National Board of Helath and Welfare.

TOKUHATA, G.K. (1969). Morbidity and mortality among offspring of breast cancer mothers. *Am. J. Epidemiol.*, **89**, 139-150.

VLAHAKIS, G., HESTON, W.E. & CHOPRA, H.L. (1977). Transmission of mammary tumour virus in mouse strain DD: further support for the uniqueness of strain $GR_1$. *J. Natl Cancer Inst.*, **59**, 1553-1555.

WALKER, A.M. (1991). *Observation and Inference*. An introduction to the methods of epidemiology. Chestnut Hill, MA: Epidemiology Resources, 73-86.